# Alpha-Synuclein in Neurodegeneration: From Shared Biology to Disease-Specific Phenotypes

**DOI:** 10.3390/cells15050451

**Published:** 2026-03-03

**Authors:** Feifei Su, Woojin S. Kim, Glenda M. Halliday, YuHong Fu

**Affiliations:** Brain and Mind Centre & Faculty of Medicine and Health School of Medical Sciences, The University of Sydney, Camperdown, NSW 2050, Australia; feifei.su@sydney.edu.au (F.S.); woojin.kim@sydney.edu.au (W.S.K.); glenda.halliday@sydney.edu.au (G.M.H.)

**Keywords:** alpha-synuclein, neuron, glia, post-translational modification, seed amplification assay, pathology modeling, Parkinson’s disease, Parkinson’s disease dementia, dementia with Lewy bodies, multiple system atrophy

## Abstract

Alpha-synuclein (αSyn) is one of the most abundant proteins in the nervous system and is currently associated with devastating synucleinopathies, yet its biology extends far beyond this. In this review, we suggest that αSyn-driven disease emerges within specific neural circuits through the combined effects of cell-type-specific roles, subcellular environments, post-translational modifications (PTMs), and co-pathology. These interacting and additive dimensions, rather than αSyn alone, generate the pathological diversity, shaping whether pathology manifests as Parkinson’s disease (PD), Parkinson’s disease dementia (PDD), dementia with Lewy bodies (DLB), multiple system atrophy (MSA), or mixed dementia phenotypes. We integrate recent advances on the physiological roles of αSyn in neurons and glia (astrocytes, oligodendrocytes, and microglia), its compartment-dependent (e.g., synaptic and nuclear) functions, and the molecular transitions (e.g., mediated by pS129) that convert functional assemblies into pathogenic conformers. Building on this foundation, we outline mechanisms through which these factors contribute to disease-specific vulnerability, progression, and clinical heterogeneity. Finally, we highlight how this multidimensional perspective on αSyn biology can inform the development of next-generation biomarkers that support precision therapies across distinct disorders.

## 1. Introduction

**Figure 1:**
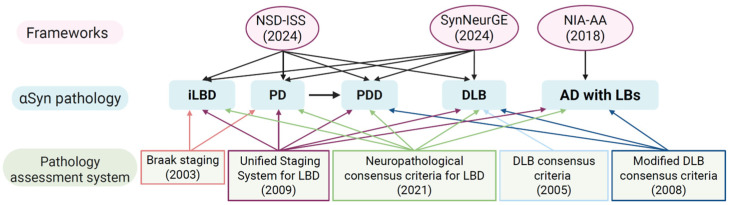
Conceptual relationships among synucleinopathy entities, pathological assessment systems, and contemporary biological frameworks. Incidental Lewy body disease (iLBD), Parkinson’s disease (PD), Parkinson’s disease dementia (PDD), and dementia with Lewy bodies (DLB) are a clinicopathological spectrum of Lewy body disorders, with Alzheimer’s disease with Lewy bodies (AD with LBs) represented as a mixed-pathology condition. Classical pathology assessment systems, including Braak staging [10], the Unified Staging System for Lewy Body Disorders [11], DLB consensus criteria [12,13], and the neuropathological consensus criteria for Lewy pathology [14], offer overlapping but non-equivalent frameworks for evaluating α-synuclein (αSyn) pathology. More recent biological and integrative models (NIA-AA [9]; NSD-ISS [6,7]; SynNeurGe [8]) emphasize disease continua, underlying biological substrates, and co-pathology rather than rigid diagnostic boundaries. Arrows denote conceptual overlap and scope of applicability. Created with BioRender.com.

Alpha-synuclein (αSyn) biology is now understood as a multidimensional process that extends well beyond the traditional framework of “synucleinopathies,” which include Parkinson’s disease (PD), Parkinson’s disease dementia (PDD), dementia with Lewy bodies (DLB), and multiple system atrophy (MSA) [1,2]. Recent advances in biomarker technologies, most notably seed amplification assays (SAAs) [3,4,5], together with emerging integrative staging and classification systems such as the neuronal αSyn disease integrated staging system (NSD-ISS) [6,7], SynNeurGe [8], and the NIA-AA [9], are shifting the field from symptom-based diagnosis toward a biology-driven understanding of disease (Figure 1).

**Beyond classic synucleinopathies**

Emerging conceptual frameworks that extend beyond traditional region-based presentation of neuronal pathology systems [10,11,12,13,14] position pathological αSyn as a central biological anchor while integrating genetic risk, co-pathology, and neuronal loss to better capture disease heterogeneity (Figure 1). This broader perspective is clinically meaningful because it situates αSyn within an interconnected biological network, allowing interpretation across conditions previously under-recognized in this context, including Alzheimer’s disease (AD) and even so-called “normal” aging [15].

Lewy pathology, for instance, is observed in 30–60% of AD cases [16,17,18,19] and in up to 30% of neurologically normal elderly individuals, in which case it is termed incidental Lewy body disease (iLBD) [20,21]. Likewise, positive αSyn detection by SAA is implicated in prodromal states such as incidental REM sleep behavior disorder (iRBD), a precursor to multiple synucleinopathies [4,22,23]. Establishing this biology-first framework is essential for understanding αSyn pathogenesis in its earliest stages, thereby enabling more definitive diagnoses and informing the development of targeted preventive interventions.

**Central question**

Despite these conceptual advances, a critical question remains: Why does αSyn pathology manifest as distinct clinicopathological entities—PD, PDD, DLB, MSA, or mixed dementia? An accompanying question is, what mechanisms determine disease-specific vulnerability and progression? Current staging and category frameworks do not fully account for cell-type-specific αSyn pathology, differentiate overlapping phenotypes such as PDD and DLB [2], or adequately address MSA. How do subcellular context and post-translational modifications (PTMs) contribute to synucleinopathies? Can a multidimensional framework integrating these factors explain clinical heterogeneity and guide precision diagnostics and therapies? This review addresses these questions by summarizing recent insights into αSyn biology and proposes a more comprehensive framework for disease-specific clinicopathology.

## 2. Physiological and Pathological Alpha-Synuclein

αSyn is a 140-amino acid protein in the synuclein family, which also includes β- and γ-synuclein [24]. As its name suggests, α-synuclein is highly conserved and abundantly expressed in the brain, with strong enrichment at presynaptic terminals [25,26,27], where most studies have focused on its role in synaptic vesicle (SV) regulation. The protein is also detected in the nucleus, and recent studies highlight its broader functions across multiple cellular compartments [26,28], an area warranting further research.

### 2.1. Physiological Role of α-Synuclein in Neurons

At presynaptic sites, αSyn interacts with components of the SV system, including the soluble *N*-ethylmaleimide-sensitive factor attachment protein receptor (SNARE) complex protein VAMP2/synaptobrevin-2, synapsins, and SV membranes [29,30]. Through these interactions, αSyn regulates key steps in SV trafficking, such as vesicle clustering [31,32,33,34,35], docking [36], and recycling pool homeostasis [31,32,33,34,35,37,38], thereby modulating neurotransmitter release. In addition to exocytosis, triple synuclein knockout mice exhibit impaired clathrin-mediated endocytosis, suggesting its role in sustaining vesicle retrieval during high synaptic activity [39].

Beyond vesicle dynamics, αSyn impacts neurotransmitter synthesis. In dopaminergic neurons, it negatively regulates dopamine production by modulating tyrosine hydroxylase (TH) expression and activity [40], where αSyn downregulation enhances TH activity and dopamine synthesis [41]. Furthermore, αSyn contributes to synaptic plasticity. Recent in vivo studies reveal that αSyn fine-tunes dopamine release by promoting release during short burst firing while attenuating it during prolonged activity, thereby adapting presynaptic output to firing patterns [42]. In hippocampal neurons, it facilitates long-term enhancement of neurotransmitter release via nitric oxide (NO)-cGMP signaling, which is essential for learning and memory [43].

Collectively, these physiological roles in presynaptic organization, vesicle cycling, neurotransmitter synthesis, and plasticity provide a mechanistic basis for understanding how αSyn dysfunction leads to synaptic impairment and the pathogenesis of Lewy body diseases (the umbrella term for neuron predominant synucleinopathies, including PD, PDD, and DLB).

### 2.2. Endogenous α-Synuclein in Glial Cells Under Physiological Conditions

While αSyn is predominantly neuronal, its presence in glial cells under pathological conditions is well established, accumulating in astrocytes and microglia in PD and in oligodendrocytes in MSA, where it forms distinct disease-specific inclusions [44,45]. However, whether αSyn is expressed in glia under normal physiological conditions remains inconclusive.

**Figure 2:**
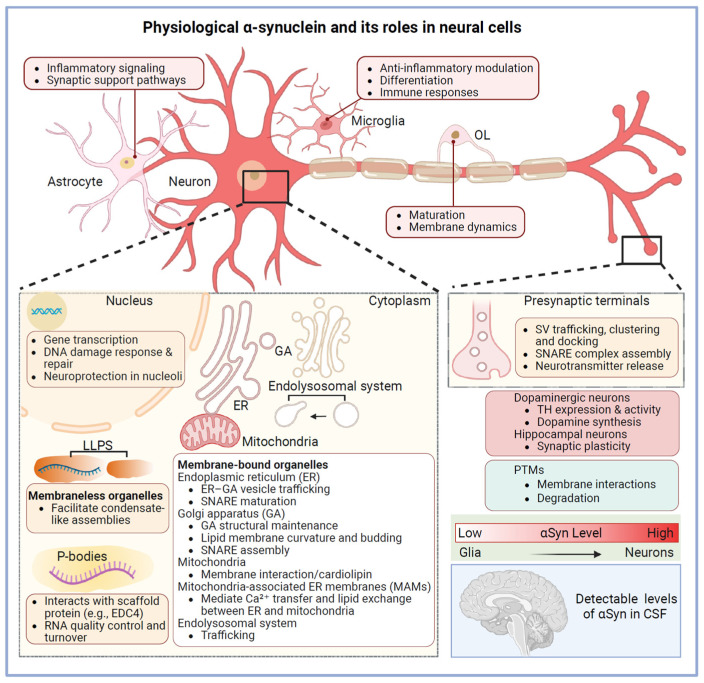
Physiological function of α-synuclein. Schematic illustration of the physiological roles of α-synuclein (αSyn) across neural cell types and subcellular compartments. αSyn is highly expressed in neurons compared with glia (representative expression levels across major brain cell types, based on human cortical data from brainrnaseq.org). In neurons, αSyn performs multiple physiological functions; in astrocytes, it contributes to inflammatory and synaptic support pathways; in microglia, it modulates immune signaling; and during oligodendrocyte (OL) maturation, it participates in membrane dynamics. At the subcellular level, αSyn functions in multiple compartments: in the nucleus, it influences gene transcription and DNA damage responses; in membraneless organelles, it interacts with processing bodies (P-bodies) and is influenced by liquid–liquid phase separation (LLPS) dynamics; and in membrane-bound organelles, αSyn dynamically associates with the endoplasmic reticulum (ER), Golgi apparatus (GA), mitochondria, and mitochondria-associated ER membranes (MAMs). At presynaptic terminals, αSyn regulates soluble *N*-ethylmaleimide-sensitive factor attachment protein receptor (SNARE) complex assembly and synaptic transmission. Post-translational modifications (PTMs) modulate αSyn membrane interactions and degradation. Under physiological conditions, αSyn levels can be detectable in cerebrospinal fluid (CSF). Created with BioRender.com.

Early studies reported only trace amounts of αSyn in cultured astrocytic cell lines detectable at both mRNA and protein levels [46]. Recent single-cell transcriptomic analyses confirm low *SNCA* expression in astrocytes and microglia in the healthy brain [47]. Immunohistochemistry (IHC) with proteinase K and formic acid pretreatment reveals low levels of αSyn in white matter astrocytes [48], while immuno-electron microscopy shows its distribution within astrocytic somata and processes, associated with subcellular organelles [48]. Functionally, astrocytes and microglia actively internalize and degrade αSyn under normal conditions, supporting protein clearance and homeostasis (Figure 2). Glial αSyn may also influence inflammatory signaling and synaptic support pathways [49] (Figure 2). In microglia, αSyn modulates anti-inflammatory responses through ERK interaction and coordinated ERK–NF-κB–PPARγ pathway activation [50]. Interestingly, αSyn is also implicated in microglial differentiation and immune responses [51]. Overall, its low basal expression suggests that most αSyn detected in these cells during disease likely originates from uptake rather than endogenous synthesis.

By contrast, oligodendrocytes exhibit low but consistent endogenous αSyn expression throughout their lineage under physiological conditions. This expression is developmentally regulated, diffuse, and non-aggregated, with enrichment in precursor and immature oligodendrocytes, implying roles in membrane dynamics [52]. This physiological pattern stands in sharp contrast to the glial cytoplasmic inclusions (GCIs) that define MSA [53,54]. Notably, GCIs emerge before overt neuronal loss, supporting the view that primary oligodendroglial dysfunction is a key driver of MSA pathogenesis [55], separating it from Lewy body disorders. Endogenous αSyn within oligodendrocytes may therefore represent the source of misfolded αSyn that accumulates in MSA [53,54].

Together, these observations underscore the cell-type–specific physiological roles and vulnerabilities to αSyn, shaping how pathology is initiated and propagated, and determining whether disease manifests as neuronal αSyn pathology in Lewy body disorders or oligodendroglial αSyn pathology in MSA.

### 2.3. Subcellular Localization and Organellar Biology

αSyn consists of three structurally and functionally distinct regions: an N-terminal domain (residues 1–60), a central hydrophobic non-amyloid-β component (NAC, residues 61–95), and a highly acidic, intrinsically disordered C-terminal domain (residues 96–140) [56,57,58,59,60]. The first 1–100 amino acids of αSyn contain seven imperfect 11-residue amphipathic repeats with the KTKGEV consensus motif. Upon membrane binding, these repeats adopt an α-helical conformation, enabling αSyn to function as an amphipathic lipid-binding protein [61,62,63].

#### 2.3.1. Endogenous α-Synuclein at Presynaptic Membranes

αSyn shows strong affinity for presynaptic membranes, driven by its preferential binding to negatively charged phospholipids through combined electrostatic and hydrophobic forces [62,64,65,66]. At the presynaptic terminal, αSyn associates primarily with the inner leaflet of the plasma membrane (PM), particularly within cholesterol-rich lipid raft microdomains [36]. Disease-associated mutations that disrupt αSyn–lipid raft interactions lead to its mislocalization away from synaptic terminals, highlighting the importance of membrane binding for proper subcellular distribution [67]. αSyn is also recognized as a “curvature sensing” protein, exhibiting a strong preference for highly curved, negatively charged membranes [68,69,70], making SVs ideal binding substrates [68,71]. Through its N-terminus-mediated membrane binding and C-terminus interactions with SV-associated proteins such as VAMP2 and CSPα [33], αSyn is proposed to facilitate SV docking and promote SNARE complex assembly at the presynaptic PM [72]. Structural studies suggest that this function is enabled by a “broken” α-helical conformation [68], composed of helix-1 (residues 3–38) and helix-2 (residues 46–93), connected by a flexible linker (residues 39–45) [73], which allows αSyn to bridge SVs and the PM. Consistent with this model, biochemical fractionation studies show that αSyn is enriched on PM-associated docked vesicles relative to undocked vesicles in synaptosomal preparations [29].

#### 2.3.2. Endogenous α-Synuclein in the Nucleus

The name “α-synuclein” reflects its initial identification at both synapses and the nuclear envelope in *Torpedo californica* [26]. Subsequent work has provided substantial evidence for nuclear αSyn and its functional relevance in vitro and in vivo [74,75]. In transgenic mice, phosphorylation at serine 129 (pS129) promotes nuclear localization, suggesting that PTMs may regulate its subcellular distribution and nuclear functions [76]. In human brain tissue, αSyn has been detected in neuronal nuclei by IHC after formic acid treatment in both control and DLB cases, and in the isolated nuclear fraction by Western blotting [77]. Within the nucleus, αSyn interacts with DNA [78,79] and histones [80], influencing gene transcription [80,81] and participating in DNA damage response and repair pathways [28,79].

Glial nuclear inclusions (GNIs) [82,83,84], although less frequent than GCIs, are a distinguishing feature of MSA from Lewy pathology. GNI is composed of filamentous aggregations of αSyn [85,86]. Nuclear neuronal inclusions (NNIs) in MSA are characterized by intranuclear accumulation of αSyn, typically detected with antibodies against the C-terminal region (residues 98–115) and pS129 [87,88]. Experimental evidence indicates that cytoplasmic αSyn fibrils can penetrate the nuclear envelope and enter the nucleus, a process linked to compromised nuclear architecture, including disruption of lamin integrity [89]. Nuclear vulnerability may represent a shared pathogenic route across affected cell types [52,85,89]. Furthermore, analyses of preclinical MSA cases have revealed αSyn accumulations within and adjacent to the nuclear membrane, suggesting that nuclear αSyn pathology may play a significant role in the early stages of MSA [85].

#### 2.3.3. Endogenous α-Synuclein at Other Organellar Membranes

Increasing evidence indicates that αSyn engages a broad array of cellular membranes, and these interactions likely contribute to both its physiological roles and pathological behavior. Beyond SVs, αSyn associates with multiple intracellular organelles, including mitochondria, the endoplasmic reticulum (ER), the Golgi apparatus (GA), and the endolysosomal system.

αSyn binds the inner mitochondrial membrane [90,91,92], via its N-terminal region, with residues 1–32 [93] or 1–25 [60] implicated in this interaction. This binding is driven by the high cardiolipin content of mitochondrial membranes, which provides a favorable negatively charged environment [94,95]. Pathological accumulation or aggregation of αSyn disrupts mitochondrial homeostasis, affecting mitochondrial dynamics [96], promoting fragmentation [96,97,98], and impairing degradation pathways [98].

αSyn localizes to mitochondria-associated ER membranes (MAMs), specialized contact sites that mediate Ca^2+^ transfer and lipid exchange between the ER and mitochondria [99,100]. Accumulation of αSyn at MAMs enhances ER–mitochondria coupling and perturbs Ca^2+^ homeostasis, linking mitochondrial dysfunction to ER stress [101,102]. Additionally, αSyn regulates the early secretory pathway by modulating SNARE–dependent ER–Golgi vesicle fusion. Disruption of this function impairs ER-to-Golgi trafficking and contributes to Golgi dysfunction [102,103,104]. Accumulated αSyn may further interfere with hydrolase trafficking at the cis-Golgi by aberrantly binding the scaffold protein GM130, thereby promoting lysosomal dysfunction [105].

αSyn also engages the endolysosomal system [106]. Intracellular αSyn aggregation has been observed within LAMP1-positive lysosomes, implicating lysosomal compartments in αSyn turnover and degradation [103,107]. Extracellular αSyn can be internalized via clathrin-mediated endocytosis and subsequently trafficked through multiple endosomal compartments. Following uptake, αSyn is sorted into Rab4A-positive fast recycling endosomes, Rab5A-positive early endosomes, Rab7-positive late endosomes, and Rab11-positive slow recycling endosomes, illustrating the complexity of its intracellular trafficking routes [108,109,110,111].

#### 2.3.4. Endogenous α-Synuclein in Membraneless Condensates

αSyn can partition into several membraneless organelles, including processing bodies (P-bodies) [112] and the nucleolus [28] (see more details in Section 2.4). Under cellular stress, αSyn can be recruited to stress granules, supporting a role in translational control and adaptive stress responses [113,114]. These compartments form through liquid–liquid phase separation (LLPS), and αSyn is now being actively examined for its behavior within these biomolecular condensates. Within these crowded and dynamic environments, αSyn may shift from its physiological state toward early condensate-like assemblies, a process proposed to facilitate the transition to solid fibril formation [115,116,117]. Consistent with this, in vitro studies show that αSyn undergoes LLPS to form dynamic liquid droplets that gradually mature into pathogenic aggregates, with early droplets containing mostly monomeric αSyn (~90%) and later stages marked by declining monomer levels, accumulating fibrils, and steady state oligomeric intermediates [118].

Collectively, these observations show that αSyn occupies a remarkably broad and dynamic subcellular landscape, engaging both membrane-bound organelles and phase-separated condensates to support essential aspects of cellular homeostasis. While its interactions with classical membranes are well established, its involvement in phase-separated compartments remains comparatively new and less studied, yet may offer important clues to early pathogenic mechanisms.

### 2.4. Alpha-Synuclein in RNA Biology

The roles of αSyn in RNA biology have become one of the most exciting shifts in the field. It has been identified as an RNA-binding protein [112,119] and interacts with RNA, other RNA-binding proteins, and RNA granules (such as P-bodies or stress granules), with implications for both biological function and neurodegeneration.

In the cytoplasm, RNA and proteins can undergo LLPS via multivalent macromolecular interactions, thereby forming dynamic, membraneless compartments [119,120]. LLPS drives the assembly of ribonucleoprotein organelles enriched in RNA and RNA-binding proteins, including nucleoli, Cajal bodies, and diverse cytoplasmic RNA granules [113,121]. The N-terminal region of αSyn mediates its interaction with P-body components, particularly scaffold protein EDC4 [112]. In PD, pathological accumulation of αSyn disrupts P-body homeostasis, leading to impaired mRNA decay and widespread alterations in neuronal gene expression [112]. Beyond canonical ribonucleoprotein granules, emerging evidence indicates that specific RNA secondary structures, such as G-quadruplexes, can directly scaffold αSyn aggregation via its N-terminal region in neurons, further reinforcing the mechanistic interplay between RNA architecture and αSyn pathology [122]. Additionally, RNA accelerates αSyn fibrillization and becomes increasingly sequestered within aggregates, especially C-terminally truncated variants with higher nucleic acid affinity, suggesting that αSyn–RNA contacts facilitate amyloid assembly despite an unresolved structural mechanism [123].

### 2.5. States of α-Synuclein

Extensive research has established the remarkable conformational plasticity of αSyn, a property central to its physiological functions. Under basal conditions, αSyn exists predominantly as a highly disordered, soluble monomer in the cell. Upon binding to lipid membranes, the first ~100 residues adopt ordered α-helical conformations [124]. These membrane-induced structural transitions enable αSyn to reversibly cycle between cytosolic and membrane-bound states while maintaining cellular homeostasis [30].

Recent liposome studies show that αSyn membrane binding involves a self-limiting multimerization process that typically traps ~six monomers per membrane-associated assembly [125]. These multimers undergo dynamic monomer exchange, which becomes spatially restricted under physiological conditions. The number and distribution of membrane-binding sites are dictated by lipid-packing defects shaped by membrane curvature and composition [126,127,128].

Importantly, physiological multimers are distinct from pathological oligomers. Physiological multimers, including tetramers, are reversible, membrane-associated, and resistant to aggregation [125]. In contrast, pathological oligomers are β-sheet-rich, membrane-disruptive, and neurotoxic [129,130]. Consistent with this distinction, membrane-bound αSyn is generally protective against aggregation, whereas the soluble cytosolic pool is more vulnerable to misfolding [131,132].

### 2.6. Post-Translational Modifications of α-Synuclein

Although often overlooked, PTMs play a pivotal role in regulating αSyn structural and functional plasticity. A wide range of PTMs, including acetylation, ubiquitination, SUMOylation, phosphorylation, nitration, and truncation, occur across the N-terminal, NAC, and C-terminal regions [44,133,134,135,136]. Under physiological conditions, αSyn exhibits a limited and tightly regulated PTM profile. N-terminally acetylated (NTA) is one of the most abundant endogenous PTMs in human brain tissue [137] and critically modulates membrane interactions [60,132,138,139]. NTA-αSyn displays enhanced affinity for membranes, particularly neutral lipids [127,138]. Ubiquitination provides a reversible regulatory mechanism that governs αSyn turnover and aggregation dynamics. Modification of lysine residues, such as K45, K58, and K60, may act as a quality-control checkpoint, sequestering membrane-damaging species and targeting cytosolic αSyn for degradation [140]. SUMOylation has similarly been proposed to stabilize soluble conformers and reduce toxic oligomer formation, although its physiological relevance remains under investigation [141]. C-terminal truncation also occurs physiologically and is not exclusively pathological [142,143]. Basal phosphorylation levels are low, with ~4% of αSyn phosphorylated at S129 in normal rat brain, although this site is highly sensitive to post-mortem dephosphorylation [144]. Additional phosphorylation sites, including Y39, S87, and Y125 (pY39, pS87, and pY125), are detectable in the soluble fractions of normal brain tissue [137,145].

The N-terminal domain is essential for membrane binding and α-helical formation, and notably, 35 of 52 identified PTMs localize to this region [137]. A recent study using a phosphomimetic mutation (Y39E) mouse model demonstrated reduced membrane interaction and increased soluble αSyn oligomers in midbrain fractions [146]. Phosphorylation at Y39 appears to impair membrane affinity by disrupting helix 2 and introducing electrostatic repulsion away from negatively charged lipids, thereby accelerating αSyn aggregation [147,148,149,150]. In contrast, nitration at the same residue alters membrane binding by steric and structural perturbation of the N-terminal helical architecture, disrupting helix packing [151]. Nitration additionally facilitates dityrosine cross-link formation, which can stabilize oligomeric assemblies and produce fibrils [151,152]. Within the NAC region, pS87 disrupts membrane binding and alters the aggregation propensity [136,153]. More than 90% of αSyn within Lewy bodies (LBs) is pS129, and 10–30% is C-terminally truncated [142,143]. This is because C-terminal truncation removes the acidic tail that normally restrains aggregation, which produces β-sheet-rich species with high seeding efficiency and further modifies fibril structure after formation [154,155]. C-terminally truncated αSyn is also present in MSA. However, current evidence indicates that its abundance is both region- and case-dependent, with some MSA cases showing a markedly higher proportion of C-terminally truncated species [155]. LC–MS/MS analyses revealed shared and disease-specific PTM signatures across synucleinopathies [137]. This supports the idea that, rather than functioning solely as disease markers, PTMs actively sculpt protein conformations and drive synucleinopathy pathogenesis.

## 3. Unified and Divergent Pathways of Alpha-Synuclein-Driven Neurodegeneration

**Figure 3:**
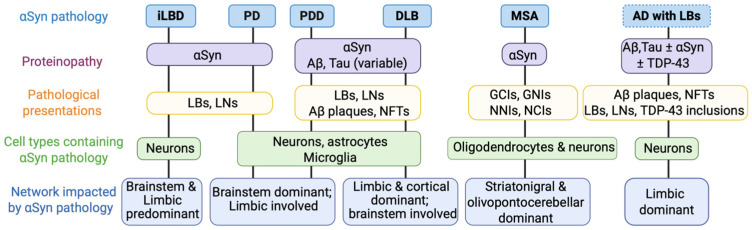
Comparative α-synuclein pathology across different disorders. The schematic summarizes how multidimensional α-synuclein (αSyn) pathology differs in synucleinopathies and Alzheimer’s disease (AD) with Lewy bodies (LBs). Preclinical Lewy body disorders, incidental Lewy body disease (iLBD), and clinical phenotypes (Parkinson’s disease (PD), Parkinson’s disease dementia (PDD), and dementia with Lewy bodies (DLB)) contain characteristic neuronal LBs and neurites (LNs). Multiple system atrophy (MSA) is characterized by αSyn within oligodendrocytes, forming glial cytoplasmic inclusions (GCIs) and glial nuclear inclusions (GNIs), as well as neuronal cytoplasmic inclusions (NCIs) and neuronal nuclear inclusions (NNIs). AD with LBs represents a mixed-protein disorder dominated by amyloid-β (Aβ) plaques and neurofibrillary tangles (NFTs). Created with BioRender.com.

Increasing evidence indicates that Lewy pathology is best conceptualized as a network-level disease that reflects this clinical phenotypic variance in Lewy body disorders. In contrast, MSA represents a distinct synucleinopathy in which misfolded αSyn predominantly accumulates within oligodendrocytes, with degeneration of vulnerable neuronal populations within specific circuits [89]. This results in a characteristic pattern of network vulnerability and system-level failure that is mechanistically distinct from that in Lewy body disorders (Figure 3).

### 3.1. Lewy Pathology as a Network-Embedded Synucleinopathy in Lewy Body Diseases

Two complementary trajectories, “body-first” and “brain-first” Lewy body diseases, have been proposed [156,157,158,159], yet both converge on the principle that αSyn pathology spreads non-randomly, following defined anatomical and functional connections. Neurons acting as network hubs with long-range projection nodes are disproportionately affected. Experimental studies highlight a cortical propagation route that begins with layer V projection neurons and subsequently engages layer II/III neurons, revealing layer-specific vulnerabilities in the cortex [160,161]. Site-specific αSyn seeding further demonstrates that the anatomical origin of pathology dictates distinct dendritic, synaptic, and circuit-level susceptibilities, underscoring that synucleinopathies are shaped by network context rather than aggregate load alone [162].

#### 3.1.1. Selective Neuronal Vulnerability: Intrinsic Properties That Amplify Risk

Across Lewy body disorders, several neuronal populations exhibit distinct vulnerability to pathological αSyn. These include early-affected autonomic neurons of the enteric and peripheral nervous systems and brainstem autonomic nuclei [163], dopaminergic neurons in the substantia nigra pars compacta [164,165], noradrenergic neurons of the locus coeruleus [166], cholinergic neurons of the basal forebrain [167,168], and glutamatergic neurons in the basal ganglia, cortex, and hippocampus [169,170]. Despite their anatomical diversity, these neurons share intrinsic features that impose substantial energetic and proteostatic stress on the cellular machinery in high metabolic demand [171], extensive axonal arborization [172,173], autonomous pacemaking activity, or sustained intracellular Ca^2+^ load [174]. When challenged by pathological αSyn, these properties heighten susceptibility to mitochondrial dysfunction, oxidative stress, impaired trafficking, and proteasomal overload, predisposing these neurons to early functional decline and degeneration [172,175,176].

#### 3.1.2. Propagation Dynamics: Prion-like Spread Across Connected Circuits

The trans-neuronal propagation of misfolded αSyn aligns with a prion-like mechanism, prompting the development of computational models to formalize disease spread. These models differ in their biological specificity but collectively reinforce the concept that pathology progression is constrained by network architecture [177]. The Network Diffusion Model (NDM) conceptualizes passive diffusion driven by gradient pathology concentration along the structural connectivity [178]. Although NDM successfully recapitulates macroscopic disease patterns, it lacks biological specificity. The Epidemic Spreading Model (ESM) was introduced to address these limitations by incorporating protein production, clearance, and host responses, thereby capturing immune-mediated reactions to abnormal protein deposition and linking these processes to disease progression [177,179]. The more recent agent-based epidemic spreading model explicitly simulates prion-like seeding and removal dynamics but remains computationally intensive and challenging to parameterize in human studies [180]. Together, these models demonstrate that selective neuronal vulnerability emerges from the interplay between intrinsic cellular susceptibility and network embedding, while clinical heterogeneity reflects differences in seeding location, propagation routes, and connectome topology [177,181].

#### 3.1.3. Divergent Clinical Phenotypes Shaped by Network Context

Phenotypic divergence among these Lewy body disorders reflects differences in the spatiotemporal distribution of αSyn pathology, the burden of co-pathologies, and network-specific vulnerability [2,162,182]. In PD, pathology predominantly affects the brainstem and nigrostriatal circuits, with limited or late cortical involvement [183]. In PDD, prolonged disease duration permits progressive invasion of limbic and associative cortical networks, superimposed on ongoing nigrostriatal degeneration, resulting in delayed cognitive decline [2]. By contrast, DLB is characterized by early and widespread cortical and cholinergic involvement, often accompanied by co-pathologies of amyloid-β (Aβ) and tau that accelerate cognitive impairment [2,184].

These patterns (Figure 3) support a model in which PD, PDD, and DLB represent overlapping yet distinct manifestations of a shared network-embedded synucleinopathy. Clinical phenotype is determined not simply by aggregate burden but by the timing, anatomical origin, and network context of αSyn engagement within selectively vulnerable neuronal populations [182,185].

#### 3.1.4. Preclinical Network Context

ILBD is widely regarded as a preclinical stage of PD or DLB [21,186]. Restricted and brainstem-predominant iLBD cases correspond to Braak Lewy pathology stages 1–3, a distribution that aligns with what is considered a preclinical PD-type form of Lewy body disease. However, a subset displays more widespread limbic or neocortical involvement, suggesting that some individuals with iLBD may be in a preclinical stage of DLB rather than exclusively preclinical PD [21,187]. In brainstem-predominant iLBD, early nigrostriatal abnormalities are already present, including reduced dopamine levels, deficits in vesicular monoamine transport, and lower striatal TH expression, changes that fall between those seen in healthy individuals and those in patients with clinically diagnosed PD [188,189]. Electrophysiological studies show that iLBD also exhibits disrupted neural network activity even in the absence of clinical symptoms, reinforcing its status as a functional preclinical stage of Lewy body disorders [190]. Transcriptomic studies indicate that PD-vulnerable brain regions possess intrinsic gene-expression patterns that are already altered in iLBD, supporting an early molecular phase of preclinical Lewy body disorders [191].

### 3.2. Co-Pathology in Lewy Body Disorders

Lewy body disorders frequently coexist with other age-related neurodegenerative pathologies, most notably Aβ and tau. This convergence reflects a complex biological interplay in which αSyn, Aβ, and tau influence one another’s aggregation, propagation, and network-level effects. In DLB, αSyn pathology is primary and widespread, typically involving neocortical, limbic, and brainstem regions early, and although Aβ and tau burdens are generally lower than in AD, they are higher than in PDD, and their frequent co-occurrence strongly modulates clinical severity, cognitive decline, and disease heterogeneity [192]. Clinically, DLB remains challenging to diagnose, with misdiagnosis rates approaching 50% in early-onset cases, most often mistaken for AD [193], with definitive diagnosis relying on post-mortem neuropathological evaluation [194]. Survival interval in DLB is shorter than in AD, underscoring the aggressive nature of αSyn-driven copathologies [195].

#### 3.2.1. Molecular Crosstalk Between α-Synuclein, Aβ, and Tau in Lewy Body Disorders

A defining feature of PDD and DLB is that αSyn pathology does not act in isolation. Instead, αSyn engages in dynamic, reciprocal interactions with Aβ and tau, forming a pathogenic interplay that amplifies neurodegeneration. Experimental and neuropathological studies show that Aβ and tau can enhance αSyn misfolding and seeding, while αSyn can accelerate Aβ and tau aggregation [17,196,197]. This tri-protein synergy disrupts proteostasis, promotes neuronal dysfunction, and helps explain why mixed pathology in DLB is associated with faster decline and more severe cognitive and neuropsychiatric symptoms. Notably, αSyn pathology in the amygdala exhibits distinct immunohistochemical and biochemical signatures in DLB with AD-pathology, pointing to disease-specific conformational heterogeneity [198].

Evidence from AD further reinforces this molecular crosstalk. Neuropathological studies show that 30–60% of AD cases harbor αSyn pathology, typically concentrated in the amygdala and other limbic regions [16,17,18,19]. These limbic-predominant deposits suggested that AD pathology-laden environments may act as permissive sites for initiating or amplifying αSyn aggregation. In addition, SAA detects aggregation-competent αSyn in 20–40% of patients with clinically diagnosed AD, confirming that αSyn co-pathology is both common and biologically active [17].

#### 3.2.2. Network-Level and Clinical Consequences of αSyn–Aβ–Tau Synergy

From a network-level perspective, synergistic interactions among αSyn, Aβ, and tau accelerate neurodegeneration more than any single pathology alone [199]. At the molecular level, these proteins exhibit prion-like seeding across anatomically connected brain regions and engage in cross-seeding, whereby misfolded assemblies of one protein promote the misfolding of others, amplifying toxicity and hastening disease progression [200,201]. Immunohistochemical studies show that αSyn deposition in AD preferentially overlaps with Aβ-rich cortical territories and, to a lesser extent, with tau-affected regions. This pattern suggests that Aβ-vulnerable networks provide a permissive substrate for secondary αSyn pathology, contributing to mixed AD–Lewy body phenotypes.

Clinically, the presence of Lewy pathology in AD is associated with worse cognition, faster decline, and a higher frequency of neuropsychiatric symptoms such as depression and hallucinations compared with “pure” AD [18,202,203]. In DLB, coexisting Aβ and tau pathology shifts the clinical presentation toward earlier memory impairment and more rapid cognitive deterioration, whereas individuals with low AD biomarker burden tend to show more prominent core Lewy body features. These observations indicate that co-pathology not only accelerates disease progression in Lewy body dementias but also shapes the clinical phenotype [204].

### 3.3. Network Vulnerability and Oligodendroglial α-Synuclein Pathology in MSA

Although oligodendroglial inclusions are the pathological hallmark of MSA, neuronal degeneration is more extensive than the neuronal loss observed in Lewy body disorders, even though Lewy body disorders have more obvious neuronal pathologies. Immunohistochemical studies show widespread GCI distribution across the MSA brain, whereas neuronal loss is more anatomically restricted (e.g., striatonigral degeneration and olivopontocerebellar atrophy) [87]. In contrast to neuronal loss in MSA, oligodendrocyte number appears relatively preserved [205,206,207]. Neuroimaging studies further revealed widespread voxel-wise atrophy that disproportionately affects both white matter and gray matter in the cerebellum and brainstem [208].

The distinctive biology of oligodendrocytes—their specialized cellular environment, their support of long-range myelinated axons, and their integration into large-scale white matter networks—has led to the recent view of MSA as a network-embedded oligodendroglial synucleinopathy. However, this framework does not fully explain why the longest corticospinal tracts are not the earliest or most severely affected, whereas cerebellar peduncles show the earliest and selective vulnerability [208]. Emerging evidence suggests that NNIs in MSA may be particularly toxic [89] and that the pronounced cerebellar and brainstem atrophy reflects combined neuronal and fiber-tract degeneration. These observations raise the possibility that rapid neuronal loss associated with nuclear αSyn pathology impacts oligodendrocyte function or that oligodendrocyte dysfunction influences neuronal survival in these regions. Overall, dysfunctional myelination alone seems insufficient to account for the pattern of degeneration.

The following subsections outline the hypothesized mechanisms of αSyn pathology in MSA and highlight unresolved questions arising from these network-level and region-specific vulnerabilities.

#### 3.3.1. Mechanistic Questions Surrounding Oligodendrocyte α-Synuclein Pathology

In MSA, the neuronal inclusions appear less regionally widespread than GCIs, a pattern that may reflect the rapid loss of the vulnerable neurons, limiting the transmission of pathological αSyn. In addition, oligodendrocytes lack the robust proteostatic and phagocytic machinery of those in microglia and astrocytes, which may contribute to the broader distribution of GCIs. It remains unclear whether oligodendrocytes initiate αSyn pathology at multiple sites due to systemic deficiencies in cellular machinery, whether neuron-oligo mechanisms drive αSyn spread, or whether other pathways enable glia-to-glia transmission. The coexistence of neuronal and glial inclusions therefore remains a central unresolved question: what is the cellular origin of αSyn in MSA?

#### 3.3.2. Hypotheses on the Origins of α-Synuclein Pathology in MSA

- Neuron-to-glia transfer hypothesis

One major hypothesis proposes that pathological αSyn originates in neurons and is subsequently transferred to oligodendrocytes. Under this model, neurons release misfolded αSyn species via exocytosis, exosomes, or synaptic leakage, which are then internalized by oligodendrocytes [89,209]. Because oligodendrocytes possess limited proteostatic capacity to handle αSyn, internalized aggregates accumulate and seed GCI formation. This hypothesis is supported by the fact that neurons express αSyn abundantly, whereas oligodendrocytes express it at low physiological levels, suggesting that neurons are a plausible source of pathogenic αSyn [53]. However, unlike PD, MSA is not known to be associated with *SNCA* mutations or multiplications. Although *SNCA* polymorphisms may modulate αSyn expression in oligodendrocytes, the absence of classical PD-linked mutations suggests a distinct pathogenic mechanism in MSA [210,211], casting doubt on this hypothesis.

- The oligodendrogliopathy first hypothesis

A recent view argues that primary oligodendroglial dysfunction precedes and drives neuronal degeneration in MSA [212]. Supporting this, αSyn has been detected in isolated oligodendrocytes from neonatal wild-type mice and from MSA patient tissue, suggesting that oligodendrocytes may be intrinsically capable of accumulating αSyn [53,213]. A key mechanistic insight comes from the mislocation of oligodendrocyte-specific phosphoprotein TPPP/p25α, which relocates from myelin to the cell soma in MSA [86]. In these diseased oligodendrocytes, p25α strongly promotes αSyn misfolding and aggregation, showing high permissiveness for pathogenic αSyn conformers [85,214,215]. Additional proteostastic failure may be driven by impaired autophagy–lysosome function and ubiquitin–proteasome stress in oligodendrocytes [216]. In addition, oligodendrocytes, which are specialized for high-throughput myelin production, are uniquely vulnerable to disruptions in protein and lipid homeostasis [217]. Accumulation of αSyn within oligodendrocytes disrupts myelin integrity, partly through altered lipid composition, including increased monounsaturated fatty acids, thereby compromising white matter function [218,219].

- The membraneless organelle hypothesis of MSA

An alternative possibility is that MSA reflects a fundamental disturbance in αSyn-related membraneless organelles. This hypothesis could account for the presence of neuronal nuclear αSyn pathology and the prominent oligodendroglial involvement. Oligodendrocytes rely heavily on RNA transport and local translation to sustain remote myelin synthesis [220]. MBP, CAII, Tau, and MOBP are all produced locally after their untranslated mRNAs are trafficked to distal myelin sheaths, associated with RNA transport granules. Disruption of αSyn-dependent phase-separation processes could therefore impair RNA handling, local translation, or the assembly of ribonucleoprotein granules in both neurons and oligodendrocytes. Such a mechanism would unify the emergence of neuronal nuclear inclusions with widespread oligodendroglial pathology and may help explain why MSA exhibits features that cannot be fully accounted for by demyelination or oligodendrocyte dysfunction alone.

#### 3.3.3. Molecular Specificity: MSA-Associated α-Synuclein Strains

Structural and biophysical analyses demonstrate that MSA-derived αSyn aggregates are enriched in β-sheet content and exhibit greater toxicity and protease resistance than PD-derived aggregates [221]. Cryo-EM studies further reveal that αSyn filaments isolated from MSA brains adopt unique asymmetric protofilament architectures [222]. Additional work highlights the molecular heterogeneity of αSyn aggregates in MSA, including less pS129 [223] but abundant PTMs within the NAC domain [137] and C-terminal truncations [155].

## 4. Targeting Alpha-Synuclein: Biomarkers and Mechanisms

αSyn biomarkers have become central to efforts to improve diagnostic accuracy, patient stratification, and therapeutic monitoring across synucleinopathies. However, the field remains challenged by the biological complexity of αSyn itself, its diverse conformations, cell-type-specific roles, and strain-dependent pathogenicity. Traditional assays that quantify total αSyn provide limited insight into this molecular heterogeneity, while next-generation approaches increasingly focus on detecting the diverse pathogenic assemblies, seeding activity, and strain-specific signatures. Together, these evolving biomarker strategies reflect a broader shift toward mechanistic, biology-driven diagnostics capable of capturing the multidimensional nature of αSyn pathology.

### 4.1. Current Biomarker Modalities

The substantial overlap in clinical features and neuropathology among synucleinopathies complicates accurate diagnosis. Because disease progression involves intercellular transfer of misfolded αSyn among brain cells, αSyn is released into the extracellular space and becomes detectable in CSF, blood, saliva, and tears [224]. Among these, CSF remains the most reliable matrix, given its proximity to the CNS and reduced influence from peripheral αSyn sources [225,226].

#### 4.1.1. αSyn Detection: Total, Oligomeric, and Phosphorylated Forms

The conventional approach to quantifying peripheral αSyn has relied on ELISA-based detection, which is easy to implement but is limited by its sensitivity and dynamic range. High-throughput proteomic platforms such as aptamer-based assays (SomaLogic’s SomaScan) [227] now enable far more sensitive, multiplexed measurement of αSyn and related biomarkers from peripheral samples. Although these technologies are advancing biomarker discovery and improving resolution of disease-associated protein signatures, their application to αSyn detection remains relatively limited.

Altered CSF αSyn levels have been reported in PD, but total αSyn is highly susceptible to blood contamination, leading to variable results across studies [228,229,230]. In contrast, oligomeric αSyn is more consistently elevated, and the oligomeric-to-total αSyn ratio improves discrimination between PD and controls [231,232]. Meanwhile, the pathological relevance of pS129 αSyn and its detectability in CSF support its potential as a biomarker [233]. However, CSF pS129 does not consistently differ between PD and controls, and longitudinal studies show that neither absolute pS129 nor the pS129-to-total αSyn ratio reflects disease presence or progression [234]. Moreover, pS129 levels do not correlate with αSyn seeding activity, limiting its diagnostic value [235]. Generally, measurements of total, oligomeric, and pS129 αSyn fail to reliably differentiate PD, DLB, MSA, and other neurodegenerative disorders [226,229,236], and diagnostic accuracy remains suboptimal for both oligomeric and pS129 αSyn detection in CSF [236].

#### 4.1.2. Seed Amplification Assays

SAAs, including real-time quaking-induced conversion (RT-QuIC), have emerged as promising tools for detecting misfolded αSyn with high accuracy in distinguishing synucleinopathies from tauopathies [237]. Interestingly, a recent study revealed that 5-34% cases are SAA-negative when the cohort comprises both sporadic and genetic PD cases [238]. While SAA-positive patients exhibit greater dopaminergic deficits, SAA-negative patients have more subcortical atrophy [238]. The SAA results in MSA remain inconsistent, with some studies reporting minimal or no seeding activity, even with ultra-sensitive protocols [239]. While these discrepancies highlight strain- and disease-specific limitations of current SAA platforms, when MSA αSyn aggregates are used as seeds, the amplified products faithfully preserve the biological and structural characteristics of the original brain-derived aggregates [240].

#### 4.1.3. Other Assays and Combined Application

Recognition that αSyn pathology extends beyond body fluids to peripheral tissues has driven the development of alternative biomarker strategies targeting salivary glands, olfactory mucosa, the gastrointestinal tract, and skin [224,241,242,243]. Among these, skin biopsy has emerged as a highly accurate, minimally invasive, and readily accessible approach, enabling both single and longitudinal sampling for the diagnosis of PD and related synucleinopathies [244,245]. Skin biopsies enable direct detection of pathological αSyn within autonomic and somatosensory dermal nerve fibers, most commonly through immunostaining for pS129 αSyn, as well as conformation-selective antibodies targeting oligomeric αSyn (e.g., ASyO5) and aggregated αSyn species (e.g., 5G4), providing complementary information on αSyn burden and aggregation state [246,247,248]. In parallel, proximity ligation assay (PLA) has been applied to skin tissue to selectively amplify oligomeric and conformationally altered αSyn, improving pathological enrichment over conventional immunostaining while preserving cellular localization [249,250]. More recently, RT-QuIC performed on skin biopsies has demonstrated high sensitivity and specificity for PD and DLB, with pathological αSyn detectable at prodromal stages such as iRBD [249,251,252,253]. Immunostaining, PLA, and SAA provide complementary readouts of peripheral αSyn pathology. In MSA, increased intraneural PLA signal supports distinct, oligodendroglial-dominant αSyn biology [249,254].

Though emerging biomarkers and methodologies bring promise, diagnostic challenges in distinguishing synucleinopathies remain. Growing evidence indicates that αSyn exhibits distinct cell-type- and disease-specific features. This paradigm shift underscores the need for future biomarkers that capture features from PTMs, conformation, seeding, and cellular context, rather than relying solely on total protein abundance.

### 4.2. Emerging Priorities in Biomarker Development

SAA has shown some capacity to discriminate disease-specific αSyn strains in PD and MSA [4,221,239]. Distinct amplification profiles were identified: type 1 patterns in samples containing LBs and type 2 patterns in samples enriched for GCIs, which mirror the underlying cellular environments. Thus, a negative SAA result does not exclude αSyn pathology, particularly in MSA. Alternatively, the reduced sensitivity of SAA in the CSF of MSA may reflect the presence of non-neuronal, oligodendroglial αSyn species that are inefficiently amplified by assays optimized for Lewy body–typed strains [221,239,255]. Given that some αSyn seeds are more toxic in MSA, the lack of seeding in SAA may result from certain oligomeric αSyn species.

In addition, samples with mixed pathologies show greater variability in SAA readouts and may even inhibit amplification, indicating the need for rigorous pre-analytical handling and quantitative assay standardization [256]. This also implicates the importance of understanding the fluid chemical interactions among αSyn, Aβ, and tau/pTau.

Accumulating evidence indicates that αSyn exists as a spectrum of conformations and species with distinct biochemical properties, cellular tropisms, and pathogenic potentials. One of the major challenges in biomarker development is therefore discriminating physiological αSyn multimers from disease-associated oligomeric species. Increasing emphasis is placed on assays targeting oligomeric and conformationally distinct αSyn species, which are considered the primary neurotoxic forms [257]. Oligomer-selective immunoassays and PLA have been explored [258], but PLA detects molecular proximity rather than pathogenic structure, meaning it cannot inherently differentiate physiological from pathological assemblies [259,260]. Therefore, the ideal antibody in PLA is one that is highly specific pathologically and abundant in the oligomeric vs. the physiological state.

### 4.3. Alpha-Synuclein Interactors as Targets for Precision Disease Profiling

**Figure 4:**
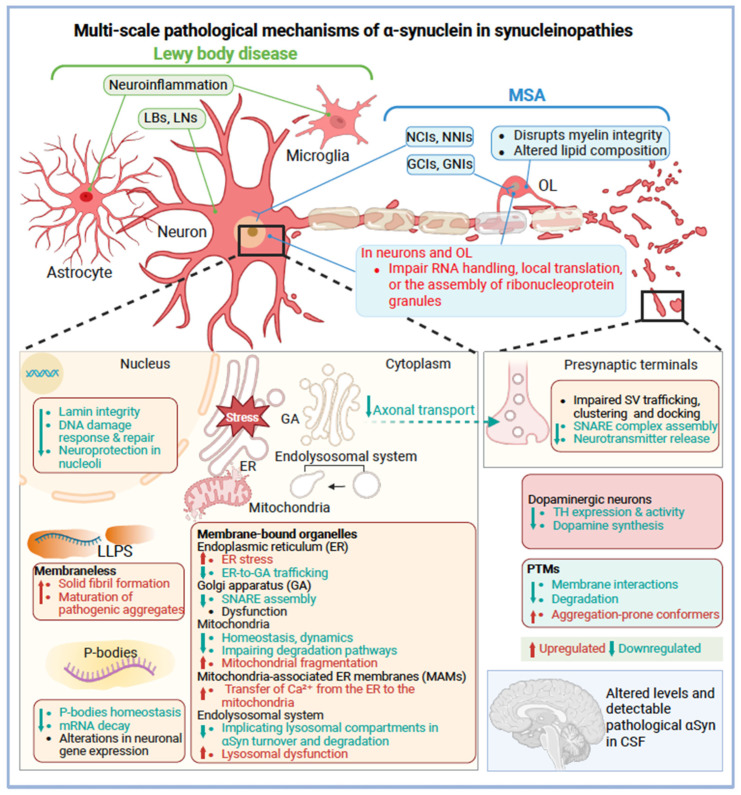
Multi-scale pathological mechanisms of α-synuclein in synucleinopathies. Schematic overview of α-synuclein (αSyn) pathology in Lewy body diseases and multiple system atrophy (MSA), highlighting cell-type–specific involvement in neurons, astrocytes, microglia, and oligodendrocytes (OLs). In Lewy body diseases, neuronal αSyn aggregation forms Lewy bodies (LBs) and Lewy neurites (LNs), which is accompanied by neuroinflammation. In MSA, oligodendroglial cytoplasmic inclusions (GCIs) disrupt myelin integrity and occur alongside neuronal cytoplasmic/nuclear inclusions (NCIs/NNIs), as well as alterations in lipid and RNA homeostasis. At the subcellular level, pathological αSyn perturbs multiple organelles and RNA regulatory compartments, including nuclear stress pathways, ER–Golgi trafficking, mitochondrial dynamics, ER–mitochondria Ca^2+^ signaling, and endolysosomal degradation. Disruption of liquid–liquid phase separation (LLPS) and P-body homeostasis impairs mRNA decay and local translation. At presynaptic terminals, αSyn disrupts synaptic vesicle (SV) trafficking and soluble *N*-ethylmaleimide-sensitive factor attachment protein receptor (SNARE) complex assembly, leading to reduced neurotransmitter release and synaptic dysfunction. Up- and down-regulated processes are indicated in red and green arrows, respectively, and pathological conformers are detectable in cerebrospinal fluid (CSF). Created with BioRender.com.

Multiple biological dimensions of αSyn point to its multiscale pathological mechanisms across synucleinopathies (Figure 4). Defining the αSyn interactors that are shared or disease-specific will help elucidate the pathways and molecular networks underpinning each disorder, an essential step towards precision treatment. For instance, αSyn interacts with mitochondrial proteins such as TOM20 [261] and VDAC1 [262], as well as the P-body scaffold protein (e.g., EDC4), linking αSyn to oxidative stress, bioenergetic failure, and disrupted mRNA processing in PD [112]. A recent study compared αSyn interactomes between PD/DLB and MSA, using in situ proximity labeling via biotinylation by antibody recognition (BAR) [263]. Pathway enrichment analysis revealed that vesicle/SNARE-associated pathways dominated PD/DLB interactions, whereas MSA was enriched in metabolic/catabolic, iron, and cellular oxidant detoxification. Notably, two of the three MSA-differentially abundant proteins (CBR1 and CRYAB) are highly expressed in oligodendrocytes, supporting their glial pathology features. A network of 26 proteins, including neuronal-specific proteins such as SYNGR3, with HSPA8 at the core, was shared between MSA and DLB/PD. Extracellular exosome pathways were consistently enriched, regardless of BAR target epitope (pS129 or aa 118–123 of αSyn) [263]. Interestingly, BAR-SYN1 (aa 91–99 of αSyn) showed no difference between DLB and healthy controls [264]. Together, these findings indicate that synucleinopathy phenotypes arise not only from αSyn aggregation itself but also from distinct, disease-specific interactomes that reflect cell-type-specific pathways. Importantly, epitopes of αSyn that remain unaffected (“innocent” epitopes) may not be prioritized as biomarkers and pharmacological targets, as they are unlikely to improve differential diagnosis and are preserved for essential biological functions. In contrast, cell-type-specific biomarkers hold greater promise for enhancing target selection and refining disease stratification.

## 5. Summary and Future Directions

Recent research increasingly indicates that the clinical heterogeneity of αSyn-driven disorders cannot be explained solely by anatomical spread. Instead, disease arises from the interplay of multiple biological dimensions, including cell type, subcellular milieu, PTMs, strain diversity, and co-pathologies such as Aβ and tau, which collectively shape how αSyn behaves within specific networks. These interacting factors influence not only where pathology develops, but how toxic it becomes, how it propagates, and why clinical trajectories diverge across PD, PDD, DLB, MSA, and mixed phenotypes.

This perspective argues for a shift toward an αSyn biology–based nosology grounded in both cell-type specificity and network context, reflected in biomarker profiles that capture such pathogenic activity. Diagnostic tools should therefore be viewed as sampling distinct nodes within a distributed pathological network rather than as isolated readouts. Integrating complementary biomarkers [265,266] within a network-aligned framework that reflects cell-type dysfunctions enables more accurate disease assessment and more refined patient stratification. Ultimately, embracing this multidimensional view together with disease-specific αSyn interactors will support the development of αSyn-targeted clinical trials that account for underlying biological heterogeneity, a prerequisite for achieving precision therapies.

## Data Availability

No new data were created or analyzed in this study.

## Abbreviations

The following abbreviations are used in this manuscript:

αSyn	Alpha-synuclein
AD	Alzheimer’s disease
Aβ	Amyloid-β
BBB	Blood–brain barrier
BAR	Biotinylation by antibody recognition
CSF	Cerebrospinal fluid
DLB	Dementia with Lewy bodies
ER	Endoplasmic reticulum
ESM	Epidemic Spreading Model
GCIs	Glial cytoplasmic inclusions
GA	Golgi apparatus
GNIs	Glial nuclear inclusions
iLBD	Incidental Lewy body disease
iRBD	Incidental REM sleep behavior disorder
IHC	Immunohistochemistry
LBs	Lewy bodies
LNs	Lewy neurites
LLPS	Liquid–liquid phase separation
MSA	Multiple system atrophy
MAMs	Mitochondria-associated ER membranes
NAC	Non-amyloid-β component
NDM	Network Diffusion Model
NTA	N-terminally acetylated
NCIs	Neuronal cytoplasmic inclusions
NNIs	Neuronal nuclear inclusions
NSD-ISS	Neuronal αSyn disease integrated staging system
OL	Oligodendrocyte
pS129	Phosphorylation at serine 129
P-bodies	Processing bodies
PD	Parkinson’s disease
PDD	Parkinson’s disease dementia
PTM	Post-translational modification
PLA	Proximity ligation assay
PM	Plasma membrane
RT-QuIC	Real-time quaking-induced conversion
SAA	Seed amplification assay
SNARE	Soluble *N*-ethylmaleimide-sensitive factor attachment protein receptor
SV	Synaptic vesicle
TH	Tyrosine hydroxylase
